# Health-Related Conditions and Depression in Elderly Mexican American and Non-Hispanic White Residents of a United States-Mexico Border County: Moderating Effects of Educational Attainment

**DOI:** 10.1155/2011/908536

**Published:** 2011-08-15

**Authors:** David F. Briones, Peter L. Heller, Luis M. Carcoba, Henry W. Weisman, Elizabeth M. Ledger, Michael A. Escamilla

**Affiliations:** ^1^Department of Psychiatry, Paul L. Foster School of Medicine, Texas Tech University Health Sciences Center, El Paso, TX 79905, USA; ^2^Department of Psychiatry and Center for Excellence in Neurosciences, Paul L. Foster School of Medicine, Texas Tech University Health Sciences Center, El Paso, TX 79905, USA

## Abstract

We investigated the prevalence of “high” levels of depressive symptomatology and 13 health-related medical conditions in elderly Mexican American (MA) and non-Hispanic white (NHW) residents of El Paso County, Texas. We analyzed the extent to which depressive symptoms in this population are associated with these conditions. Elderly MA residents possessed a higher prevalence of current depression, a relatively unique health-related condition profile, and were more likely to experience a set of conditions that impede participation in daily life—conditions that we found to be strongly associated with high depressive symptomatology in the elderly. After adjusting for educational attainment, using multiple regression analyses, depression was not associated with ethnicity and only six of the health related conditions showed significant differences between MA and NHW subjects. We believe these results provide an important insight into the mechanism of health-related conditions and depressive symptomatology in a large sample of elderly MAs; and how conditions typically attributed to MA ethnicity may in actuality be an artifact of socioeconomic status variables such as educational-attainment.

## 1. Introduction

Over the past 15 years or so a number of studies have investigated the prevalence of depression in Hispanic populations [1–22], along with comorbidity between depression and various types of health-related conditions [10, 17]. A number of authors feel that assessment of depression and comorbid diseases among elderly minority populations has been relatively neglected in the geriatric psychiatry literature [3, 10, 17, 23] and that this state of affairs specifically applies to Mexican American (MA) elderly populations, where depressive symptomatology and diseases such as diabetes are relatively common [24]. In actuality a fair number of such studies have been conducted, and we feel that attention should now be focused on precisely what factors are associated with these differential ethnic findings. A salient issue for us involves the extent to which these previous findings may actually reflect life conditions associated with socioeconomic status (SES) per se rather than some yet to be determined ethnic or cultural trait. Comorbidity between depressive symptomatology and various types of health-related conditions does represent a crucial research problem because negative health outcomes, including untimely death, tend to be associated with depression [25–28]. The underlying factors behind these negative outcomes remain largely unknown. However, since ethnic groups as a whole may exhibit disparities at the level of income and education, important determinants of ethnic differences in rates of depressive symptomatology may be linked to relatively independent factors associated with SES.

**Rates of Depression and Hispanic Ethnicity**

Prevalence rates for “high” depressive symptomatology among elderly Hispanic populations vary widely from study to study, from a high of 26.7% among elderly Hispanics residing in the Los Angeles area [21] to a low of 11.4% reported in the San Luis Valley Health and Aging Study [16]. However, virtually all Hispanic versus non-Hispanic white (NHW) comparisons reveal significantly higher rates of depression in the Hispanic population, including the well-known five-state Hispanic Established Populations for Epidemiologic Study of the Elderly (H-EPESE) [17].

**Health-Related Conditions and Depression**

The most thorough study of the relationship between self-reported health-related conditions and depression was published by Black and her colleagues [17]. However, because the H-EPESE sample did not include a NHW comparison sample, H-EPESE findings were compared with those of the New Haven EPESE [29]. This methodology is quite legitimate because all EPESEs share a basic set of questions and all use multistage sampling. However it is not known to what extent regional differences in ethnic group location may influence comparative research findings. More recently, Romero and her colleagues [10] evaluated the rates of depressive symptoms in Hispanic and NHW elderly residents of Bernalillo County, New Mexico and found higher rates of depressive symptoms in the Hispanic group. However, their logistic regression analyses found that these differences in depressive symptoms could largely be explained by education and income differences between the two groups.

We intend to enhance this line of research by presenting research findings from a comparative sample of MA and NHW elderly residents of El Paso County, Texas. We assessed prevalence rates for “high” depression and 13 health-related conditions in a residential population of elderly MA and NHW respondents. We then compared the two ethnic groupings by calculating odds ratios (OR) for the probability of finding these conditions among the MA versus NHW elderly. Finally, we conducted a series of logistic regression analyses to ascertain the extent to which a SES variable (educational attainment) might modify our ethnic group findings.

El Paso County offers a unique opportunity for making MA versus NHW health comparisons, especially where issues related to (SES) are at stake. In no El Paso County census tract do MAs comprise a minority of the tract's overall population. Although relatively poor (25.2% of El Paso County's 2009 population live below the official poverty line) [30] the fact that MAs comprise 81.8% of the County's population [30] enables researchers to include a broader SES spectrum among MA household samples. Average years of formal schooling for the first four waves of the five southwestern state that H-EPESE sample was between 4 and 5 [31]. Although our sampling only increased this average by two years, fully 38% of our MA elderly sample possessed at least a high school degree [32].

## 2. Methods

### 2.1. Survey Participants

 We interviewed a stratified random sample of 1,152 noninstitutionalized elderly (65 years and older) residents of El Paso County, Texas, in 2000-2001. This sample represents 84 percent of respondents originally contacted for interview. The survey instrument was translated into Spanish through backtranslation for accuracy. In-home interviews were conducted in Spanish or English depending on respondents' preference. Twenty-four census tracts were randomly selected from 94 census tracts specified in the 1990 census after stratification for median income and ethnic composition. Specifically, eight census tracts were randomly selected from each of the lower, middle, and upper-middle income categories. Each of these 24 census tracts was screened through telephone interviews to identify households containing one or more members 65 years of age or older. We attempted to contact every house listed in the El Paso County Polk Directory for all 24 tracts. Up to five phone contacts were attempted for each listed household at staggered time intervals in order to develop our sampling frame. Screening interviews also provided demographic information including respondent's age, gender, and self-reported ethnicity. Independent random sampling procedures were then established for MAs and NHWs. These procedures ensured virtually equal representation, within each of the three census tract income categories, of women and men, and age cohorts 65–74 and 74+.

**Characteristics of the Sample**

Specifically, comparative sample sizes for MAs and NHWs are 799 and 353, respectively. Mean and median ages for the entire sample were 74.9 and 74.0. Mean ethnic group ages are virtually identical (MA = 74.9, NHW = 75.0); percentage age 75 or higher (MA = 48.4, NHW = 51.0); percentage female (MA = 50.7, NHW = 49.9); percentage high-school graduate or higher (MA = 35.0, NHW 86.9); percentage above median household income for entire sample (MA = 23.9, NHW = 84.3); percentage with no medical insurance (MA = 38.4, NHW = 0.8). It should be noted that we compromised some degree of randomness in order to achieve better ethnic group balance in such factors as SES, “old” versus “old-old” age categories, and gender. It should also be noted that costs related to preliminary screening did not permit inclusion of households without telephones.

### 2.2. Assessment of Depression

Depressive symptomatology was measured with the CES-D (Center for Epidemiologic Studies Depression Scale) [33], the most widely used measure of depressive symptomatology among community-dwelling Hispanics, including MA residential populations [17]. This 20-item scale has been determined reliable for elderly respondents [34] and is predictive of current and future clinical depression [35]. Each item score can range from 0 to 3 depending on the frequency with which respondent has experienced a particular symptom during the past week. Scale scores can range from 0 through 60, and a score of 16 or above was set in this study as representing a higher probability of depression [36]. A limitation of the CES-D is that it does not provide a diagnosis of depression but only measures severity of depressive symptoms. However, economic limitations of the study did not permit structured diagnostic interviews, and we thus chose a measure of depressive symptomatology that has been widely used with Hispanic populations, so that rate comparisons with previous studies would be meaningful.

### 2.3. Assessment of Health-Related Conditions

We assessed 13 health-related conditions. We asked respondents if, during the past three months, they had to cut down on things they usually do and/or if they have stayed in bed all or most of the day because of illness or injury. We also asked whether the respondent was usually troubled by shortness of breath when walking at an ordinary pace on level ground and/or whether she/he had a tremor or unusual slowness of movement where muscles have become unusually rigid, coupled with the problem of falling down when walking. Specific diseases were assessed by asking whether a doctor had told the respondent that he/she had a heart attack (coronary/myocardial infarction/coronary thrombosis), high blood pressure, diabetes (sugar in urine/high blood sugar), stroke, broken hip, cancer, or arthritis (rheumatism). We also ascertained whether each respondent had a urinary and/or bowel problem serious enough to keep him/her from doing some of the things they like to do (see Table 1). Self-reporting of medical conditions is a frequently used technique in epidemiological research. The validity of self-reports apparently depends on the quality of methodology and question wording [37]. In this case, the 13 health-related conditions were studied and our question wording is virtually identical to that used in the H-EPESE [17]. We should also note that these health-related conditions may or may not in themselves constitute causal agents; for example, cutting back on activities or being bedfast may well be resultants of other health-related conditions and diseases.

### 2.4. Assessment of Ethnicity

Ethnicity was defined through a combination of respondent's self selection and childhood and adolescent developmental history. This multifaceted procedure yielded a sample of 799 elderly MAs and 353 elderly NHWs: 414 MA respondents reported having been born in the U.S. and/or having lived in the U.S. until age 16, and/or having been reared mostly in the U.S.; 385 report being born in Mexico and/or living in Mexico until age 16 and/or mostly reared in Mexico. It is legitimate to place both of these groupings under the ethnic category, “MA”. Nevertheless, this finding demonstrates some of the diversity within the Mexican American community itself (especially along the US-Mexico border) and the danger of using a simplistic self-identity measure of ethnic membership. We employed this methodology because of our interest in defining ethnicity in terms of actual life and acculturation experiences. We thus validated our ethnic group classification through a well-accepted Mexican acculturation scale [38]. As reported elsewhere [32], our ethnic categories are strongly correlated with this scale (*r* = 0.86).

### 2.5. Assessment of Educational Attainment

Education was measured by years of educational attainment, including ordinal categories for “high-school graduate,” “college graduate,” and “at least some post college education.” A high school degree represents successful completion of 12 years of formal education after preschool, both in the U.S. and in Mexico. Educational attainment has been selected because of its importance to a number of mental health phenomena [32, 39].

### 2.6. Analyses

 The prevalence rates for “high” levels of depressive symptoms (CES-D score of 16 or higher) were calculated for respondents with and without each health-related condition; the *t*-test statistic was utilized to ascertain statistical significance (Table 1). In addition, the “condition” of ethnicity (rates of high depression in persons with and without MA ethnicity) was analyzed with the *t*-test statistic (Table 1). The same procedure was repeated (Table 2) by comparing ethnic group mean scores for each condition. In order to economize on tabular presentations only significance levels for *t*-values have been reported. Odds ratios (OR) indicate the extent to which statistically significant findings are clinically meaningful (Tables 1 and 2). ORs depicted in Tables 1 and 2 have straightforward interpretations because each OR represents findings from a 2 × 2 table in which each measure has two response categories. Finally, we ran a series of direct (all predictors simultaneously enter into the equation) two-step logistic regression analyses (see Table 3). In Step  1 the analysis predicted “high” depression and each of the 13 conditions by MA versus NHW ethnicity. Findings for ethnicity, adjusting for respondent's educational attainment, were analyzed in Step  2. Logistic analysis is indicated for multivariate analysis when the dependent variable is dichotomous (e.g., high versus low depressive symptomatology) [39]. Logistic regression also permits a mixture of dichotomous and continuous variable predictors in the same equation. For us, this two-step analysis sheds light on the extent to which ethnic effects found in Table 2 are moderated by the inclusion of one important attribute of SES.

## 3. Results

### 3.1. Prevalence Findings: Entire Sample

Table 1 presents prevalence of “high” rates of depressive symptoms for MAs, NHWs, and for the total sample of respondents suffering from each health-related condition. In this study, El Paso's elderly MAs possessed a significantly (*P* < 0.001) greater rate of “high” depressive symptoms (13.3%) than did their NHW counterparts (5.2%). The odds for “high” depression among El Paso's MA elderly are almost three times (OR = 2.7) the odds for elderly NHWs.

With three exceptions—cardiovascular disease, broken hip, and cancer—health-related conditions tend to significantly associate with depressive symptomatology among our total sample of 1152 respondents. It should also be noted that the four nonspecific health-related conditions (the necessity of cutting back on activities, staying in bed, possession of tremor/unusual movement, and shortness of breath) are by far the most strongly associated with depressive symptomatology in this sample. The odds for “high” symptomatology among respondents who have stayed in bed all/most of the day (OR = 5.0), cut back on normal activities (OR = 5.4), experienced shortness of breath during normal walking (OR = 5.2), or have tremor/unusual movement (OR = 4.7) are profoundly higher than odds for respondents who do not suffer from these conditions.

Several specific diseases also meaningfully were associated with “high” levels of depressive symptoms; the odds for “high” depressive symptoms among those who suffer from hypertension (OR = 1.8), diabetes (OR = 2.2), stroke (OR = 2.3), arthritis (OR = 2.1), urinary incontinence (OR = 2.9), and bowel incontinence (OR = 2.4) were two or more times the odds for those who did not have these conditions. Three other specific illnesses—cardiovascular disease, broken hip, and cancer—did not significantly associate with depressive symptomatology in our sample.

### 3.2. Prevalence Findings: MAs

 Prevalence rates for these 13 health-related conditions are presented for the elderly MA and NHW samples in Table 2. Also included are significance test results and odds ratios comparing MA prevalence rates with those of NHWs. Regarding the four nonspecific health-related conditions, 34% of El Paso's elderly MAs have had to cut back on activities, 25% were experiencing shortness of breath, 22% possessed tremor/unusual movement, and 19% had to stay in bed most or all of the day during the three-month period prior to interview. Additionally, 49% had been diagnosed with diabetes, 53% had hypertension, and 49% had arthritis. Finally, in descending order, Mexican American prevalence rates for urinary incontinence, cardiovascular disease, cancer, stroke, bowel incontinence, and broken hip were 18%, 14%, 10%, 10%, 8%, and 4%.

### 3.3. Odds Ratios Comparing Prevalence Rates for MAs and NHWs

Elderly Mexican Americans in El Paso were significantly more likely than their NHW counterparts to possess five of the thirteen conditions. Specifically, MA odds ratios were significantly higher for cutting back on activities (OR = 1.8), being bedridden (OR = 2.1), possessing tremor/unusual movement (OR = 2.0), diabetes (OR = 3.3), and bowel incontinence (OR = 2.4). On the other hand (and in line with other research findings) [24] elderly MAs appeared to be relatively healthier than their NHW counterparts with respect to cardiovascular disease (OR = 0.64), cancer (OR = 0.35), and arthritis (OR = 0.70). Thus the odds for cardiovascular disease among El Paso's elderly MAs were 1.6 times (1 ÷ 0.64) *lower* than those for NHWs. *Lower* odds among MAs for arthritis and cancer were (1.4 and 2.9, resp.).

### 3.4. Logistic Regression Analysis: The Moderating Effects of Educational Attainment

 A logistic regression analysis was performed in order to ascertain whether an important SES variable (educational attainment) could account for some of the differences between prevalence rates of conditions between the ethnic groups summarized in Table 2. Relative effects of MA-NHW ethnicity versus educational attainment on possession of “high” depressive symptomatology and the 13 health-related conditions are summarized in Table 3. When considered alone ethnicity results mirror those portrayed in Table 2. Elderly MAs are significantly more likely than NHWs to possess high depressive symptomatology, necessarily cut back on activities, experience tremor/unusual movement, suffer from diabetes mellitus, be bedridden, and experience bowel incontinence. On the other hand, elderly MAs were significantly *less likely* than NHWs to suffer from cardiovascular disease, arthritis, and cancer.

In the current study, “high” depressive symptomatology rates were not higher in the MA sample, after adjusting for a key socioeconomic variable (educational attainment). After including educational attainment in the equations, only diabetes and bowel incontinence were significantly more likely to occur in the MA than NHW grouping. Cardiovascular disease, arthritis, cancer, and hypertension were significantly less likely to occur among elderly MAs after adjusting for educational attainment.

## 4. Discussion

### 4.1. Importance of Direct Biethnic Comparisons in a US-Mexico Border Environment

 Studies that focus exclusively on MA populations typically draw ethnicity-based conclusions either by looking at various degrees of within-group acculturation or by comparing their research findings with those collected from NHW samples living elsewhere in the United States. By focusing on this highly-populated border county (est. 2009 population of 751,296) [40] we were able to simultaneously assess ethnic and educational effects on possession of depressive symptoms and 13 health-related conditions and to include the comparison of MA and NHW samples in the same study. The fact that El Paso County is 81.8% MA [40] means that, although relatively poor—25.2% of El Paso County's 2009 population live below the poverty line—[40] our sampling strategy permitted us to include a broader SES spectrum among our MA household sample than is typically found in other published research.

### 4.2. Depressive Symptoms in Elderly Mexican Americans in the Current Study

The current study found that 13.3% of the elderly MA sample endorsed high levels of depressive symptoms (CES-D rating of 16 or higher), which is lower than the 25.6% of elderly Hispanics who reported high rates of depressive symptoms in the 5-state H-EPESE sample [17]. However, the 13.3% rate of MAs endorsing high levels of depressive symptoms in the current study is similar to the rate previously reported in a study from New Mexico (9.5% of males and 19.2% of females reported high levels of depressive symptoms in that study) [10]. This may reflect the fact that the current study (El Paso County) and the study conducted in New Mexico (Bernalillo County) were done in populations where Hispanic population constituted a large proportion of the county. In demographic situations such as these, a sizable array of social resources may be available to MAs. Indirect evidence for this suggestion is provided by Ostir et al. in their analysis of 1993-1994 five-state H-EPESE data [41]. These authors found a −0.548 unit decrease in CES-D score for each 10% increase in Mexican American neighborhood population, a finding that may be linked to the availability of familial, friendship, and socioeconomic resources. The accessibility of such resources may tend to insulate elderly MAs from life conditions associated with minority status. This phenomenon may be especially salient in El Paso County where the MA population comprises 81.8% of the total [30]. A previous community survey of El Paso County adult residents found that MAs were significantly more familistic than their NHW counterparts [39]. Findings from that study, however, brought into question whether or not familism, as opposed to what the authors refer to as “resource-in-place” support, is a positive contributor to mental well-being.

### 4.3. Effects of SES on Depression and Health-Related Conditions that May Directly Affect Quality of Daily Life Across Ethnic Groupings

In concert with previous research findings (including above-cited sources) we found that the rate of “high” depressive symptomatology for El Paso's elderly MAs (13.3%) was significantly higher than that for their NHW counterparts (5.2%); however, when adjusting for effects of educational attainment (Table 3), no significant ethnic differences in symptomatology were found. This is consistent with the analyses of Romero and her colleagues [10] recently conducted in a community sample from New Mexico. 

The current study found a number of nonspecific and specific health-related conditions to be more typical of the MA sample, but, as with depressive symptomatology, many of these differences became nonsignificant after adjusting for educational attainment. 

In the sample as a whole—all subjects regardless of ethnicity—several medical conditions (diabetes, arthritis, hypertension, and stroke) were significantly associated with higher depressive symptomatology in bivariate analyses (Table 1). Subjects who reported tremors/unusual movements and/or shortness of breath also showed significant proneness towards high levels of depressive symptoms. These illnesses or medical symptoms thus reflect risk factors for higher depressive symptomatology among El Paso County's elderly population. The mechanisms of this association may reflect as yet undetermined causal patterns or a common biological pathogenesis of depression and some of these disorders. 

Respondents who had had to cut back on activities and/or spent much of the day in bed possessed odds for high levels of depressive symptoms at least five times the odds for those who did not so endorse these items (Table 1). This association may be due to the direct effects of depression, however, on phenomena such as anhedonia and fatigue. 

Our findings with regard to differences between MA and NHWs are especially instructive, as they demonstrate that many differences in rates of illness between these groups may actually be an artifact of differential SES of the two ethnic groups. Despite our rigorous attempts to stratify our sampling procedures in order to include a greater number of upper-middle-class MAs, the characteristics of our MA sample speak for themselves. The MA versus NHW disparity in percentages of high school graduates in our sample was 35% to 86.9%. Only 23.9% of MAs in our sample possessed incomes above the El Paso median; 38.4% of these elderly MAs were without health insurance, including MEDICARE. This set of predicaments associated with lower class status may well lead to “high” depression regardless of ethnic identification. It should also be noted that hard physical labor associated with many working-class jobs makes physical injury highly probable and brings workers in close proximity to potentially toxic chemicals. Such conditions may in themselves constitute risk factors for mental health, various diseases, and other health-related conditions.

### 4.4. MA Ethnicity and Rates of Medical Illnesses

 After controlling for socioeconomic factors, the current study did find several significant differences in the “prevalence” of medical diseases between elderly MAs and NHWs. Mexican American elderly were *more prone *to report having diabetes and bowel incontinence compared to NHW subjects (adjusting for educational attainment), while cardiovascular disease, arthritis, hypertension, and cancer were *less *likely to be reported in the MA grouping. The specific reasons for these differences in self-reported disease/health condition rates in our sample could be due to a number of factors. For instance, knowledge of some diagnoses (cardiovascular disease, hypertension, cancer) requires specific diagnostic procedures or may be caught in regular checkups, and there may be ethnic or cultural differences in how often persons see doctors or participate in health screenings. There may also be specific genetic factors, diet/nutritional factors, environmental factors, cultural, and psychological factors which contribute to increased risk or protection from particular diseases [42–44].

### 4.5. Limitation

 Our study is focused on a single geographic region and county in which specific factors may decrease applicability to MAs in other regions and to other Hispanic populations. The current study also focused on depressive symptoms as measured by the CES-D and did not include formal diagnoses of depression and other mental disorders. A final limitation is that rates of medical illnesses were based on self-report and not actual diagnostic tests. Nevertheless, we believe that these results provide an important insight into the mechanisms of chronic disease/other health-related conditions and depression in a large sample of MAs and offer an important new data set not contained in previous studies of the MA elderly. Furthermore, this study gives a unique insight into how these illnesses present in a major United States metropolitan area in which MAs are the cultural majority. 

### 4.6. The Need for Additional Research

 This cross-sectional study has revealed a number of findings that both corroborate and augment those previously reported in the MA elderly mental health literature. Nevertheless, a number of questions remain unanswered that could be better addressed through longitudinal research. *The first question* deals with the disparity of reported prevalence rates for depression that exists between elderly MA and NHW populations. Specifically what types of social and community resources are the most salient contributors to mental well-being? To what extent is mental well-being related to the differential availability and accessibility of such resources? To what extent does the proportion of MAs in a community/metropolitan area affect the availability of these resources? *The second question* involves the temporal sequence that occurs between depression, the onset of specific medical illnesses, and how particular depressive or medical symptoms affect the quality of life of the person. *Thirdly*, we suggest that future research address (1) the extent to which multiethnic disease/health-related condition profiles exist within societal populations, (2) the extent to which such profiles associate with differential rates of depression, and (3) the extent to which these profiles and “high” depression rates are moderated by effects of SES. Carefully designed studies will be needed in order to determine whether particular ethnic and cultural groupings do indeed have specific risk or protective factors for psychiatric and medical diseases and, if these factors exist, to identify them.

## Figures and Tables

**Table 1:** Prevalence of high depressive symptomatology^a^ by self-reported health-related condition for total elderly sample (*N* = 1152).

		Prevalence (%)						Prevalence (%)			
		High depressive symptoms^a^						High depressive symptoms^a^			
Condition	*N*	With condition	Without condition	Odds ratio	95% CI	Condition	*N*	With condition	Without condition	Odds ratio	95% CI
	
											
All CES-D respondents	1152	10.7				Ethnicity (Mexican Am)	799	13.3	5.2***	2.66	1.58 4.47
Cut back activities	347	22.2	5.0***	5.40	3.59 8.10	Stayed in bed	185	27.0	6.9***	5.00	3.32 7.53
Tremor/unusual movement	228	25.2	6.7***	4.70	3.16 6.99	Shortness of breath	274	24.1	5.8***	5.19	3.49 7.73
Cardiovascular disease	174	12.6	9.8	1.33	0.81 2.17	Hypertension	621	12.7	7.4**	1.84	1.23 2.74
Diabetes mellitus	284	16.5	8.2***	2.22	1.49 3.30	Stroke	121	19.0	9.2***	2.31	1.40 3.81
Broken hip	53	11.3	10.2	1.12	0.47 2.68	Cancer	173	8.1	10.6	0.74	0.41 1.33
Arthritis	586	13.5	7.0***	2.07	1.39 3.10	Urinary incontinence	195	20.5	8.1***	2.94	1.93 4.47
Bowel incontinence	79	20.3	9.6*	2.40	1.34 4.32						

^a^Based on a total CES-D score of 16 or higher.

*P < 0.05; **P < 0.01; ***P < 0.001.

**Table 2:** Prevalence of health-related conditions for elderly Mexican Americans (*N* = 799) and Non-Hispanic whites (*N* = 353) with odds ratios^a^.

	Prevalence of condition					Prevalence of condition			
Condition	MA%	NHW%	Odds ratio	95% CI	Condition	MA%	NHW%	Odds ratio	95% CI
Cut back activities	33.8***	22.6	1.75	1.31 2.34	Stayed in bed	18.9***	10.0	2.10	1.42 3.11
Tremor/Unusual movement	22.4***	12.5	2.02	1.41 2.89	Shortness of breath	24.9	21.9	1.18	0.87 1.59
Cardiovascular disease	13.5*	19.4	0.64	0.46 0.90	Hypertension	52.9	56.3	0.88	0.68 1.13
Diabetes mellitus	30.5***	11.7	3.31	2.31 4.47	Stroke	10.3	11.1	0.92	0.61 1.37
Broken hip	4.3	5.4	0.78	0.44 1.39	Cancer	10.5***	25.3	0.35	0.25 0.48
Arthritis	48.5**	57.4	0.70	0.54 0.90	Urinary incontinence	17.9	14.8	1.26	0.89 1.78
Bowel incontinence	8.3***	3.7	2.36	1.28 4.34					

^a^Odds ratios greater than 1 signify a higher Mexican American prevalence.

*P < 0.05; **P < 0.01; ***P < 0.001.

**Table 3:** Logistic regression analysis predicting “high” depressive symptomatology and 13 health-related conditions by Mexican American (*N* = 799) versus non-Hispanic White (*N* = 353) ethnicity, adjusting for educational attainment^a^.

	B MA versus NHW	B adjusting for educational attainment		B MA versus NHW	B adjusting for educational attainment
Depression (“high”)	0.98***	0.43	Stayed in bed	0.74***	0.13
Cut back activities	0.56***	0.12	Shortness of breath	0.17	−0.13
Tremor/unusual movement	0.70***	0.27	Hypertension	−0.13	−0.33*
Cardiovascular disease	−0.44*	−0.54*	Stroke	−0.09	−0.42
Diabetes mellitus	1.20***	0.94***	Cancer	−1.06***	−0.72***
Broken hip	−0.25	−0.36	Urinary incontinence	0.23	0.04
Arthritis	−0.36**	−0.46**	Bowel incontinence	0.86**	1.22***

^a^Educational attainment (*after adjusting for MA versus NHW ethnicity*) significantly and positively associated with “high” depressive symptomatology (*P* < 0.001), cutting back on activities (*P* < 0.000), tremor/unusual movement (*P* < 0.001), diabetes mellitus (*P* < 0.05), being bedridden (*P* < 0.001), shortness of breath (*P* < 0.01), hypertension (*P* < 0.05), stroke (*P* < 0.05), cancer (*P* < 0.05), and bowel incontinence (*P* < 0.05). Education did not significantly associate with cardiovascular disease, broken hip, arthritis, or urinary incontinence (after adjusting for effects of MA versus NHW ethnicity).

*P < 0.05; **P < 0.01; ***P < 0.001.
